# Investigating the effect of inquiry-based stress reduction on mortality awareness and interpersonal problems among intensive care unit nurses

**DOI:** 10.1186/s12888-022-03764-y

**Published:** 2022-02-10

**Authors:** Soheila Tajnia, Sedigheh Iranmanesh, Neda Asadi, Mark McDermott

**Affiliations:** 1 Nursing Research Center, Kerman University of Medical Sciences, Kerman, Iran; 2 University of East London (UEL), London, UK

**Keywords:** Stress; mortality awareness, Interpersonal problems, Intensive care unit

## Abstract

**Introduction:**

Caring for dying patients is one of the job stressors. Nurses in intensive care units are among the medical staff who have a close interaction with dying patients. Studies have shown that psychological interventions are very helpful in improving thinking about death and its problems. Therefore, this study was conducted to investigate the effect of Inquiry-Based Stress Reduction on mortality awareness and interpersonal problems among intensive care unit nurses in southeastern Iran.

**Materials and methods:**

This was a Quasi-experimental study with a pretest-posttest design in southeast of Iran in 2021. Nurses were selected using the convenience sampling method and divided into intervention (*n* = 32) and control (*n* = 35) groups using the block randomization method. The intervention group received a two-hour Inquiry-Based Stress Reduction counseling session every week for 6 weeks. Data were gathered using Multidimensional Mortality Awareness Measure and Inventory of Interpersonal Problems before, immediately after, and 6 weeks after the intervention. IBM SPSS Statistics software version 25 was used for data analysis.

**Results:**

In the intervention group, the mean scores of Mortality Awareness before, immediately after, and 6 weeks after the intervention were 130.41 ± 5.91, 164.47 ± 8.66, and 163.91 ± 9.29, respectively. Therefore, in the intervention group, the increase of Mortality Awareness mean score was statistically significant (*P* < 0.001). In the control group, the mean scores of Mortality Awareness before, immediately after, and 6 weeks after intervention were 129.63 ± 5.59, 135.26 ± 11.14, and 132.66 ± 5.62, respectively. Difference between the two groups was significant (*P* < 0.001). The results also showed that in the intervention group the mean scores of Interpersonal Problems immediately after and 6 weeks after the intervention were lower than before the intervention (*P* < 0.001). In the control group, Interpersonal Problems increased over time (*P* < 0.001). Accordingly, the difference between the two groups in terms of Interpersonal Problems during the study was statistically significant (*P* < 0.001).

**Conclusion:**

The study results suggest that the Inquiry-Based Stress Reduction is an appropriate intervention method to improve mortality awareness and reduce interpersonal problems in intensive care unit nurses.

## Introduction

The Health and Safety Executive in the United States has named nursing at the top 40 stressful professions and believes that nursing is at the top of high-stress careers in the healthcare system [1]. Caring for dying patients is one of the job stressors, which due to the constant contact of nurses with these patients, they are exposed to this type of stressor [2]. It is unrealistic and unfair to expect nurses to behave appropriately when people die, regardless of nurses’s awareness of death [3]. Due to the nature of their job, nurses deal with the concept of death more than other people. In the definitions of nursing, pain relief, death with dignity, and peace are mentioned. If nurses consider death to be a frightening and sinister issue, they will not be able to deal with the death of patients calmly and effectively [4].

Also, The spread of Covid-19 has created waves of anxiety and fear around the world, including in Iran. Because there is still no definitive treatment for this disease [5], The pandemic nature of COVID-19 puts health care workers around the world in an unprecedented position and puts a high workload on them [6]. Severe respiratory problems in patients with the virus have greatly increased the need for medical care and hospitalization in intensive care units. And has caused the health care system to face many problems due to the occurrence of this disease [7]. also, mortality rates in intensive care patients with COVID-19 are very high, ranging from 6 to 86% of hospitalized patients, and the mortality rate among COVID-19 patients in the intensive care unit is 29% in the world [8]. In a study conducted in Tehran, the capital of Iran. After examining 16,000 cases, they expressed the death rate in the ICU and CCU was 62.7% of confirmed cases, 52.2% of suspected cases [9]. Meanwhile, nurses and physicians are at the forefront of the fight against this virus, suffering from anxiety, fear and, stress due to the power of transmission and the high prevalence of this disease [10].

Nurses are the first to come in contact with terminally ill and dying patients, as well as their family and friends, and spend more time providing care for them. Awareness of death and positive viewpoint and attitudes in nurses can be effective in caring for patients and supporting their family members [11]. The Dying process in Iranian culture special the Islamic view is different from other cultures. The majority of the people in Iran follow Islam, Iran is part of a group of countries where the nuclear family, based on marriage, predominates. The relationship and affection between Iranian families are very strong, so in addition to the patient, family members will be severely affected if they are told their loved one is dying [12]. In Iran, the preservation of human dignity at the time of death [13], the recitation of the Qur’an for the patient, and her purification at the time of death are done by families.

Studies reported that psychological interventions are very helpful in improving thinking about death and the resulting anxiety [14, 15]. One of these psychological interventions is the Inquiry-Based Stress Reduction (IBSR) method, which is mainly based on the work of Byron Katie. It is a self-exploration method based on experience, meditation, and mindfulness [16]. In this intervention, negative emotions are identified and the participant realizes that these thoughts are not useful for him/her and as a result, lets go of the negative feelings [17]. Although there are therapists who use IBSR in their counseling, IBSR appears to be relatively unknown [18] and has often been used to address a specific range of issues, particularly work-related stress. Individuals who have practiced this method have reported several benefits, including reducing depression, stress levels, and anger; having healthier relationships, and greater relaxation in their lives [16]. Also, among the treatments that have been considered for psychological symptoms in the last two decades is the Mindfulness-Based Stress Reduction program (MBSR) [19] and it is different from the IBSR method. In MBSR, People learn mindfulness techniques, such as mindfulness breathing (focusing on breathing and observing thoughts without getting caught up in them) and body scan (raising awareness and accepting emotions in different parts of the body) to help them understand painful emotions and Negative help and do not need to fight, suppress or prevent them from having a meaningful life and achieving goals [20].

Mindfulness training help to eliminate dysfunctional reactions and can affect interpersonal problems [21]. Leufke et al.’s findings showed that IBSR intervention significantly reduced a wide range of psychological symptoms, interpersonal sensitivity, hostility, obsession, and paranoid thoughts [17]. Smernoffa Eric et al. [22]. (2019) concluded that all subscales of anger and anxiety were significantly reduced after the IBSR intervention. Lin et al.’s (2019) finding showed that MBSR intervention, reduced stress and negative affect and increased positive affect and resilience after the MBSR counseling [23]. The results of a study by Song et al. (2021) showed that MBSR counseling was effective in reducing measures of depression, anxiety and stress, and increasing their mindful awareness in nursing students [24]. In IRAN, Shamooshaki et al.’s (2021) and Rahimi et al.’s (2014) finding showed that MBSR counseling [23] decreased nurses’ job stress [25] and Procrastination [26]. The results of various studies in this field have not been able to fully confirm or deny the impact of these interventions. For example, the results of Anderson’s study on 68 adults examining the effect of mindfulness on reducing rumination, anger, depression, and increasing concentration and attention in life showed that there was no significant difference between the intervention and control groups [27].

In general, due to the fact that nurses in intensive care units spend more time in contact with the dying and dying patients, it is necessary to use various methods, including the use of psychological interventions to assess the effectiveness of this type of intervention, mortality awareness and, their interpersonal problems. Moreover, this issue has not been considered in our country so far. Considering the aforementioned studies, in order to examine the effect of IBSR on mortality awareness and interpersonal problems among intensive care unit nurses, we decided to conduct this study.

## Materials and methods

### Design and setting

This was a quasi-experimental study. The research environments were the intensive care units of Shafa and Shahid Bahonar Hospitals in Kerman, southeast of Iran in 2021. In Iranian health context here, most nurses had a bachelor’s degree and specialized training in intensive care units who worked in the Level 3 Intensive Care Units of two governmental hospitals in Kerman, southeast Iran. These hospitals have the highest rate of patient admission to intensive care units with various diagnoses. The nurse-to-bed ratio in intensive care units is 1:2.

### Sample size and sampling

All 254 intensive care unit nurses were screened in terms of inclusion criteria(120 nurses in Shafa Hospital and 134 nurses in Shahid Bahonar Hospital). Inclusion criteria included: having at least 6 months of work experience in an intensive care unit, having at least a bachelor’s degree in nursing, no symptoms of psychosis, not using any psychological treatment before and during the intervention. Exclusion criteria included: missed more than 2 sessions of counseling. Eligible nurses were divided into intervention and control groups using the block randomization method. Based on the study of Ruth Leufke et al. [28] and also Type I error)α(= 0.05 and Type II error (β) = 0.02, The sample size in each group was estimated at 36 nurses. Considering the dropouts, the final sample size was determined 41 nurses per group. A total of 15 nurses were excluded from the study for various reasons, including COVID-19 conditions and missing sessions. Finally, 32 nurses in the intervention group and 35 nurses in the control group were studied. It should be noted that a sample size of at least 30 people is recommended for experimental research [29].

### Measures

To collect data in the present study, three questionnaires of demographic characteristics, Multidimensional Mortality Awareness Measure (MMA- M), and Inventory of Interpersonal Problems (IIP-32) were used.

### Demographic characteristics

It included age, gender, marital status, income, level of education, clinical experience, work experience in an intensive care unit, job position, type of work shift, type of intensive care unit, faces with life-threatening illness, and having the experience of seeing another person’s death.

### Multidimensional mortality awareness measure (MMA- M)

This measure was designed by Mark R. McDermott et al. in 2015 to measure mortality awareness. It consists of 36 items in five subscales: Mortality Legacy (10 items); Mortality Fearfulness (10 items); Mortality Acceptance (5 items); Mortality Disempowerment (6 items); and Mortality Disengagement (5 items) scored on a 7-point Likert scale (1 = Strongly disagree, 7 = Strongly agree). The possible maximum and minimum scores are 252 and 36. Higher scores indicate a higher mortality awareness. McDermott et al. reported its reliability with the Cronbach’s alpha coefficient for the four subscales of mortality legacy, mortality fearfulness, mortality acceptance, mortality disempowerment in a range of 0.73 to 0.87 and for the subscale of mortality disengagement 0.59 [16]. MMA-M was translated into Persian by two faculty members. Faculty members were fluent in both the main and the target language and also had a history of translating the questionnaire and were familiar with the concepts of the questionnaire. The translated versions were compared with each other and the differences and contradictions were corrected. There was no change in the questionnaire at the validity process and grammar and spelling problems were corrected. The final translation was translated back into English. The final version of the MMA-M was also approved by the original designer.

Face validity in this study, we assessed face validity qualitatively and quantitatively. In the qualitative method, the questionnaire was assessed by 10 persons from the target community, and their opinion about clarity, feasibility, readability, consistency of style, and formatting was collected. In the quantitative stage, the 36 items in the questionnaire were scored based upon a 5-point Likert scale. The Impact Score (IS) (frequency × importance) was calculated. The items were adopted if they had an impact score higher than 1.5 [30].

The content validity of the questionnaire was evaluated by ten faculty members of Kerman Universitiy of Medical Sciences. Content Validity Ratio (CVR) and Content Validity Index (CVI) were assessed. The formula was used to calculate the CVR for the total scale after the participants replied to the items. According to Lawshe’s table, an acceptable CVR value for ten specialists was 0.62. CVI assesses the relation and simplicity of an item to the content represented in the questionnaire. Both Item-level CVI (I-CVI) and Scale-level CVI (S-CVI) were calculated. The minimum score of 0.78 was considered acceptable for both I-CVI and S-CVI [31].

Reliability As the evaluation of the reliability of internal consistency alone provides no information on the stability of participants’ responses, we used internal consistency. The internal consistency of the questionnaire was assessed using Cronbach’s alpha coefficient for each dimension separately and in total. The Cronbach’s alpha coefficient was 0.70. (Translation and Cultural Adaptation Process, Wild et al. (2005)).

### The inventory of interpersonal problems (IIP-32)

This measure was designed by Barkham et al. in 1996. It consists of 32 items in eight subscales. Reliability analyses were conducted for the eight scales of the IIP-32. The alpha coefficients were as follows: H. Assertive = .86, H. Sociable = .89, H. Supportive = .75, T. Dependent = .71, T. Caring = .72, T. Aggressive = .85, H. Involved = .75, and T. Open = .80. The alpha for the IIP-32 was .86 [32]. Items are scored on a 5-point Likert scale from zero (not at all) to 4 (strongly). The maximum and minimum possible scores are 128 and 0. Higher scores indicate more interpersonal problems In the current study, the content validity index was determined to be 0.89. Also, the reliability (internal consistency) of this questionnaire was confirmed with a Cronbach’s alpha coefficient of 0.90.

### Intervention

After obtaining informed written consent and providing the necessary information about the objectives of the study, nurses completed the measures. Were both hospitals control and intervention group. After selecting the qualified nurses in the available method, they were divided into two groups of intervention and control based on random block allocation.

Then, nurses in the intervention group received a two-hour IBSR counseling session every week for 6 weeks. Participants underwent IBSR intervention as a group workshop. The intervention was given by SI who had previously been trained in this regard and ST was facilitator. With the permission of the Vice Chancellor for Health of Kerman University of Medical Sciences and better effectiveness, attendance sessions were held in Sina Hall of Shahid Bahonar Hospital with proper social distance and the use of personal protective equipment such as masks and disinfectants and proper ventilation. Again, the relevant information should be mentioned. At the end of each session, the date and time of the next session were done and announced in coordination with the samples. In order to perform the intervention, a video projector was used to display the videos. Nurses in the control group did not receive any intervention..The measures were collected again from both groups immediately after and 6 weeks after the intervention. It should be noted that the next session was at times agreed upon by the participants.

### IBSR intervention

The package in question is “Let’s love what it is” by Byron Kitty.

The IBSR consists of two parts: The first step is to write judgments about distressing living conditions, past, present and future. Conditions that make a person nervous or frightened by others or people they do not like. Stressful thoughts and beliefs are identified in a systematic and comprehensive way. Emotions are allowed to be expressed without fear or with any punitive consequences, and the participant declares, “Thinks he / she is facing a stressful situation again, a situation that is reliably stressful.” Allow the participant to mentally face the time and place of the stressful event and put the worksheet in front of them and answer the questions.

Part 2: The participant is asked to choose the main and stressful thoughts from the written ones and to examine the thoughts with 4 questions. The IBSR questions are as follows:

1. Is it true?
2. Do you know for sure that this is true?
3. How do you react, what happens when you believe that thought?
4. How would you be without that thought?

Questions 1 and 2 seek the answer “yes” or “no”. If a sample answers “no” to question 1, then question 3 is asked. By asking question 3, the participant states a list of physical behaviors and reactions that occur in the presence of a stressful thought and writes it on a worksheet. However, if a sample answers “yes” to question 1, the researcher goes to question 2: “Do you know for sure that this is true?” The participant can think in response to the above questions. After asking question 4, ask the participant to close his / her eyes and visualize himself / herself without any stressful thoughts, and ask him / her to write the answer on the worksheet. The participant is instructed to find the real answers to the four questions, without any pre-determined instructions.

After answering the IBSR questions, the participant is asked to reverse the written comments and judgments. The aim is to change the direction of the main thought so that the participant can find a new interpretation of reality. The participant is asked to change the sentence as if he were writing about himself. He is asked to find 3 real examples in which the change of direction of the main thought is equally true (for a given sentence, there are three or four opposite sentences or more). Inverted sentences are reviewed with the help of four IBSR questions. We have all the answers and reviews. There is no right or wrong answer. Let the truth rise from within and face the question. Every opinion or belief is reviewed so that it is enough for the mind to bring it to the level of consciousness and this belief is resolved.

Changing directions provides an opportunity to experience perspectives that have not previously been considered. By doing this, the participant understands and experiences that he or she should not automatically trust stressful thoughts; But he can choose different interpretations of reality and experience situations that are considered stressful with peace of mind. In this way, by discovering other perspectives, one may discover that what one does not like about another person is also true about oneself. As a result, one realizes the solutions that are simple and radical. Gains knowledge of the structure of thought in a way that one understands one’s inner cause and effect; And gradually the mind achieves silence and peace as a result of recognition; And one can accept the facts without resistance and internal war.

### Data analysis

IBM SPSS Statistics software version 25 was used for data analysis. Descriptive statistics (frequency, percentage, mean and standard deviation) were used to describe the demographic characteristics of the samples in the two groups. Independent t-test and Chi-square tests were used to determine the similarity of the two groups in terms of the demographic characteristics and confounding variables at the beginning of the study. The score of MMA-M and IIP-32 at different times (before, immediately after, and 6 weeks after the intervention) in the two groups followed the normal distribution. According to the condition of equality of variances, the parametric test of analysis of variance was used in repeated measures.

### Ethical considerations

This article is an excerpt from a master's nursing student dissertation.The ethics committee of Kerman University of Medical Sciences approved this study (IR.KMU.REC.1398.623 and NO: 98000988). It was explained to the subjects that their participation was voluntary and they could leave the study whenever they wanted. Full explanations were given about the objectives of the study and the application of its possible results. From all of the participants obtaining informed consent and they were ensured that their information would remain confidential. Participants were allowed to withdraw from the study at any desired time.

## Results

The mean age of the subjects in the intervention group was 34.88 ± 4.80 years and in the control group 33.71. ± 4.36 years. There was no significant difference between the intervention and control groups in terms of age (Table 1).

**Table 1:** Description and comparison of demographic characteristics of the subjects

Group Variable	Intervention		Control		Independent T-test	*P*-value
	Mean	Standard Division	Mean	Standard Division		
Age	34.88	4.80	33.71	4.36	1.04	0.30
	Frequency	Percentage	Frequency	Percentage	Chi-square test statistics	
Gender					1.22	0.35
Male	4	12.5	8	22.9		
Female	28	87.5	27	77.1		
Marital Status					1.14	0.31
Single	8	25.0	13	37.1		
Married	24	75.0	22	62.9		
Education					0.24	0.75
BSc	26	81.2	30	85.7		
MSc and Higher	6	18.8	5	14.3		
Income					1.33	0.33
In the range of 20–40 Million IRR	7	21.9	4	11.4		
More than 40 million IRR	25	78.1	31	88.6		
Work experience in a clinical environment					1.83	0.43
< 5 Years	6	18.7	8	22.9		
5–10 Years	10	31.3	6	17.1		
10 < Years	16	50.0	21	60.0		
Work experience in an ICU					0.11	0.89
< 5 Years	23	71.9	24	68.6		
5–10 Years	8	25.0	10	28.6		
10 < Years	1	3.1	1	2.8		
Type of work shift					1.15	0.55
Morning shift	9	28.1	13	37.1		
Evening shift	15	46.9	12	34.3		
Night shift	8	25.0	10	28.6		
Type of intensive care unit					4.76	0.06
ICU	20	62.5	30	85.7		
CCU	12	37.5	5	14.3		
Position					0.88	0.62
Nurse	31	96.9	32	91.4		
Head Nurse	1	3.1	3	8.6		
Faces with life-threatening illness					0.51	0.62
Yes	11	34.4	15	42.9		
No	21	65.6	20	57.1		
Having the experience of seeing another person’s death					0.26	0.99<
Yes	31	96.9	33	94.3		
No	1	3.1	2	5.7	1.22	

The results showed that there were no significant differences between the two intervention and control groups in terms of gender, marital status, education, income, work experience in a clinical environment, work experience in an ICU, type of work shift, type of intensive care unit, position, faces with life-threatening illness, and having the experience of seeing another person’s death. Therefore, the two groups were identical in terms of demographic variables (Table 1).

In the intervention group, the mean scores of mortality awareness before, immediately after, and 6 weeks after the intervention were 130.41 ± 5.91, 164.47 ± 8.66, and 163.91 ± 9.29, respectively. Therefore, it is clear that during the study in the intervention group, the increase of mortality awareness was statistically significant. In the control group, the mean scores of mortality awareness before, immediately after, and 6 weeks after intervention were 129.63 ± 5.59, 135.26 ± 11.14, and 132.66 ± 5.62, respectively. In the control group, mortality awareness increased over time. There was a statistically significant difference between the two groups in terms of mortality awareness during the study. The results of the Bonferroni post hoc test showed that the mean score of mortality awareness was significantly higher in the intervention group than the control group immediately after and 6 weeks after the intervention (Table 2).

**Table 2:** Comparison of the mean scores of mortality awareness of the subjects

Group Mortality Awareness	Intervention		Control		Mean Difference (Intervention-Control)	*P*-value
	Mean	Standard Division	Mean	Standard Division		
Before the intervention	130.41	5.91	129.63	5.59	0.78	0.58
Immediately after the intervention	164.47	8.66	135.26	11.14	29.21	0.001>
Six weeks after the intervention	163.91	9.29	132.66	5.62	31.25	0.001>
Source of change	Sum of Squares		Df	F	P-value	Eta^2^
Time	16,269.08	2	125.70	0.001>	0.66	16,269.08
Group ^a^ Time	9701.92	2	74.96	0.001>	0.54	9701.92
Group	20,896.31	1	337.20	0.001>	0.84	20,896.31
Error	4028.05	65				

^a^ Analysis of variance in repeated measures: Modified for multiple comparisons: Bonferroni

In the intervention group, the mean scores of interpersonal problems before, immediately after, and 6 weeks after the intervention were 107.59 ± 2.45, 67.94 ± 2.11, and 68 ± 2.99, respectively. Therefore, it is clear that during the study in the intervention group, the interpersonal problems were significantly reduced. In the control group, the mean scores of interpersonal problems before, immediately after, and 6 weeks after the intervention were 105.37 ± 6.24, 115 ± 4.47, and 114.71 ± 5.02, respectively. In the control group, interpersonal problems increased over time. There was a statistically significant difference between the two groups in terms of interpersonal problems during the study. In other words, the mean score of interpersonal problems was significantly lower in the intervention group than the control group immediately after and 6 weeks after the intervention. The results of the Bonferroni post hoc test showed that there was a statistically significant difference between the mean scores of interpersonal problems before the intervention compared to immediately after and 6 weeks after the intervention. However, there was no statistically significant difference between immediately after and 6 weeks after the intervention. In other words, despite the completion of the intervention, its effect continued until 6 weeks after the intervention. In the control group, the mean scores of interpersonal problems were significantly increased immediately after the intervention and 6 weeks after the intervention compared to before the intervention (Table 3).

**Table 3:** Comparison of mean scores of interpersonal problems of the subjects

Group Interpersonal Problems	Intervention		Control		Mean Difference (Intervention-Control)	Test Statistics^a^	*P*-value
	Mean	Standard Division	Mean	Standard Division			
Before the intervention	107.59	2.45	105.37	6.24	2.49	1.35-	0.18
Immediately after the intervention	67.94	2.11	115.00	4.47	46.89-	7.05-	0.001>
Six weeks after the intervention	68.00	2.99	114.71	5.02	47.01-	7.04-	0.001>
Test statistics^b^	2855.82		41.63				
*P*-value	0.001>		0.001>				

^a^ Mann-Whitney U test

^b^ Analysis of variance in repeated measures

## Discussion

The study aimed to investigate the effect of inquiry-based stress reduction on mortality awareness and interpersonal problems among intensive care unit nurses. According to the results of the present study, mortality awareness was significantly increased during the study in the intervention group. The results also showed that the mean score of mortality awareness was significantly higher in the intervention group immediately after and 6 weeks after the intervention. According to our review of the literature, there was no study on the effect of IBSR on mortality awareness in nurses or other populations. Therefore, to discuss and explain the purpose of the study, the studies on the effect of IBSR on other psychological problems and other similar studies were used. In this regard, in a study conducted by Krispenz et al., the results showed that in the IBSR group, chronic stress and trait anxiety were significantly reduced during 3 months. While, in the control group, there were no statistically significant changes [33]. Also, Luff et al. reported a wide range of benefits as a result of using IBSR, including having a positive effect on the therapist health and protecting against burnout, being an effective self-care method, improving self-awareness and self-compassion, making the individual more cognitively flexible, and improving metacognitive consciousness [16]. Considering that the aforementioned studies indicated the positive effect of IBSR on psychological problems and the results of the present study also showed the positive effect of this type of therapeutic intervention, the results of these studies could be considered in line with the present study. In another study, Wong indicated that death education courses had a significant and positive effect on students so that they have a more positive view of death and less fear and avoidance of facing death [34]. Niknejadi et al. in a study found that mindfulness-based cognitive-behavioral therapy reduces death anxiety [35]. Raeisi et al., also, concluded that mindfulness training in the mindfulness group improved body image and death anxiety in patients [36]. A review of the results of these studies showed that psychological interventions could affect people’s attitudes and anxiety about death, and from this perspective, they are in line with the results of the present study.

The results of the present study showed that interpersonal problems were significantly reduced during the study in the intervention group. However, in the control group, interpersonal problems increased over time. There was a statistically significant difference between the two groups in terms of interpersonal problems during the study. In other words, the mean scores of interpersonal problems were significantly lower in the intervention group immediately after and 6 weeks after the intervention than in the control group. Despite the completion of the intervention, the effect continued until 6 weeks after the intervention. Findings of Leufke et al.’s study showed that this type of intervention significantly reduced interpersonal sensitivity which remained significantly low in the 3-month follow-up evaluation [37]. Mafi et al., also, found that mindfulness-based cognitive therapy could be a general strategy to improve interpersonal problems and correct early maladaptive schemas in patients with depressive disorder [38]. These studies were considered because they emphasized the effect of educational interventions on improving interpersonal problems. Therefore, from this viewpoint, the results of these studies could be considered in line with the results of the present study. According to the results of these studies on the effect of educational interventions on interpersonal adjustment and considering that the more individuals could be adapted to their environment and people in this environment, their interpersonal problems could be reduced. The results of these studies are also in line with the results of the present study.

Interpersonal problems experienced in relation to others can cause psychological distress [32], anxiety, and psychological helplessness in an individual [21]. In the nursing profession, teamwork is one of the most important and influential factors; Because the quality of patient care depends on the cooperation of nurses, other members of the care team, patients, and even their families. One of the preconditions for such a partnership is respect, cooperation, and relationships between members in the workplace. The free exchange of information, care, and health are compromised without participatory communication, and the patient is at risk for adverse outcomes [39]. Therefore, it is necessary to provide the needed conditions for nurses to reduce interpersonal tensions and problems so that they can provide quality care. It seems that the use of psychological interventions could be a way forward in this direction. Therefore, IBSR educational intervention could be introduced as one of the effective methods. Of course, considering that no study has been done in this field so far, more research is needed for better and more definite conclusions.

This study had its limitations. Among other things, the poor cooperation of some staff in answering the questionnaires due to boredom, fatigue, high workload, etc. was considered as one of the limitations in conducting the research, which we tried to hold meetings at times agreed by the participants. Another limitation of this study was the existence of covid19 disease and the limitations of the intervention which were solved by following health protocols and holding virtual sessions.

## Conclusion

The results of the present study showed that IBSR could be effective on mortality awareness and interpersonal problems in nurses. Considering these results and the importance of mortality awareness and interpersonal problems in nurses, it is suggested to use this method of intervention to increase mortality awareness and reduce interpersonal problems in hospital staff, especially nurses, who have the most interaction with patients and their behaviors and problems have a direct and effective impact on patient care.

### Limitations

The present study, like any other study, had a number of limitations. One of the limitations of this study was the reduction of the sample size of nurses in the study. Considering Time-consuming study and covid-19 epidemic condition and increasing the workload of nurses, Out of a total of 82 nurses with inclusion criteria, A number of nurses were unable to continue attending the sessions due to the covid-19 disease, despite follow-up and were excluded from the study at the end. Therefore, it is suggested that these interventions be performed in future studies with a higher sample size.

## Data Availability

The datasets used during the current study are available from the corresponding author on reasonable request.

## Abbreviations

IBSR: Inquiry-Based Stress Reduction
IIP-32: Inventory of Interpersonal Problems
MMA-M: Multidimensional Mortality Awareness Measure
