# Correction to: Investigating the effect of inquiry-based stress reduction on mortality awareness and interpersonal problems among intensive care unit nurses

**DOI:** 10.1186/s12888-022-03882-7

**Published:** 2022-04-13

**Authors:** Soheila Tajnia, Sedigheh Iranmanesh, Neda Asadi, Mark McDermott

**Affiliations:** 1 Nursing Research Center, Kerman University of Medical Sciences, Kerman, Iran; 2 University of East London (UEL), London, UK

**Correction to: BMC Psychiatry 22, 106 (2022)**

**https://doi.org/10.1186/s12888-022-03764-y**

Following publication of the original article [1], the authors identified that the year was provided incorrectly in reference 5. The reference has been corrected.

5. Mangolian Shahrbabaki P, Dehghan M, Maazallahi M, Asadi N. Fear and anxiety in girls aged 7 to 11 years old and related factors during the coronavirus pandemic. Clin Child Psychol Psychiatry. 2022;27(1):259-68.

The original article [1] has been corrected.
